# Octogenarian Atrial Fibrillation Ablation with New NavX EnSite “Live View” Tool

**DOI:** 10.1155/2021/8834133

**Published:** 2021-02-11

**Authors:** Mariano Rillo, Zefferino Palamà, Francesco Zonno, Giulia My, Raffaele Punzi, Luigi My, Luigi Sciarra

**Affiliations:** ^1^Cardiology Unit “Villa Verde”, Taranto, Italy; ^2^Abbott Medical Italia, Sesto San Giovanni, Italy; ^3^Cardiology Unit “Policlinico Casilino”, Rome, Italy

## Abstract

**Background:**

Atrial Fibrillation ablation in older patients represents a challenge to be addressed to ensure the improvement of the quality of life and survival of these patients. New mapping system tools can help to treat older patients because of its ability to simplify and reduce procedural risks. The new NavX EnSite “Live view” tool allows dynamic “beat to beat” activation and voltage mapping visualization in order to instantly recognize vein disconnection and minimize RF deliveries.

**Methods:**

An 81-year old patient with paroxysmal AF and well-documented firing focus trigger underwent pulmonary veins isolation using NavX EnSite Precision, HD Grid multipolar catheter, and the new “Live view” tool.

**Results:**

All pulmonary veins were successfully isolated with no procedural complications. “Live view” tool allows to perform shorter and safer procedure (total procedural time: 90 minutes, left atrium dwell time: 60 minutes, total RF delivery number: 78).

**Conclusion:**

“Live view tool” allows dynamic activation and voltage mapping in order to perform a safe and tailored approach to ablation, especially in older patients.

## 1. Introduction

In recent years, we have witnessed a generational shift in patients undergoing atrial fibrillation (AF) ablation [1]. Therefore, AF ablation in older patients represents a challenge to be addressed to ensure the improvement of the quality of life and survival of these patients.

## 2. Case Presentation

**Figure 1:**
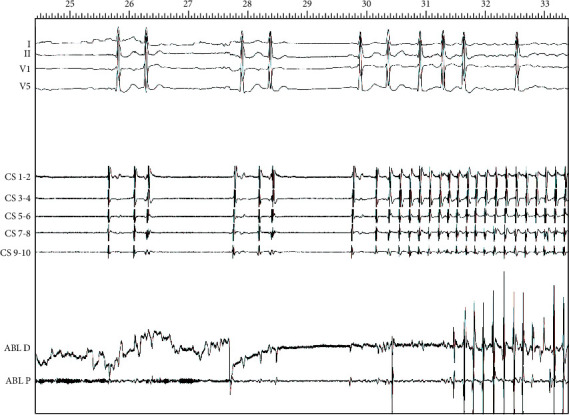
“P on T” phenomena as AF trigger during catheter positioning (I-II-V1-V6: ECG lead; CS: coronary sinus decapolar diagnostic catheter tracings; ABL: bipolar ablation catheter tracings).

We report the case of an 81-year old patient with a strongly symptomatic paroxysmal AF (mEHRA score 2b) [2], resistant to multiple drug therapy attempts, with a good functional state, and well documented to be a pulmonary vein focal firing AF (“P on T” phenomena as shown in Figure 1). The patient was highly motivated to solve his arrhythmia problem. We performed AF ablation with pulmonary veins isolation using NavX EnSite Precision, HD Grid multipolar catheter, and the new “Live view” tool that allow dynamic “beat to beat” activation and voltage mapping visualization.

**Figure 2:**
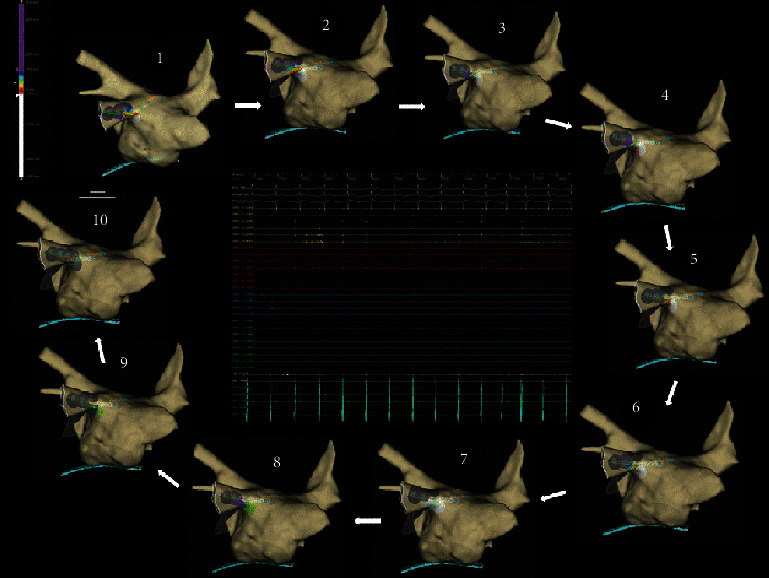
“Live view” tool allows to instantly view the presence of conductive gaps in vein isolation and their disappearance. Beat to beat activation map visualization (1 to 10), EGMs recordings show PV signal disappearance during ablation (HD grid in vein ostium during ablation).

As we can see in Figure 2, the new “Live view” tool allows to instantly view the presence of conductive gaps in vein isolation and their disappearance.

All pulmonary veins were successfully isolated, and no procedural complications were observed. Total procedural time was 90 minutes, left atrium dwell time was 60 minutes, and total RF delivery number was 78.

## 3. Discussion

A tailored ablation strategy can target specific triggers and drivers of atrial fibrillation [3]. When atrial fibrillation mechanism is well identified, as in our case, ablation procedure should not be denied only for the age factor. In fact, a successful atrial fibrillation ablation significantly and persistently improves quality of life, with a significant reduction in arrhythmia symptoms frequency and severity [4]. Key to the success of a tailored ablation strategy is an accurate identification of AF triggers and their treatment. In terms of efficacy, durable pulmonary vein isolation represents the cornerstone of any AF ablation procedure, especially when PV firing focus was identified.

The limits in terms of safety can also be overcome thanks to the use of the new tools, like the new NavX EnSite Precision “Live view” tool, that allow dynamic “beat to beat” activation and voltage mapping visualization. These features turn into a reduction of unnecessary radiofrequency deliveries, of left atrium dwell time, and of total procedural time. Indeed, this tool allows to instantly recognize vein disconnection during RF application, without having to redo a new voltage map to check vein deconnection.

A tailored approach [5], especially when the arrhythmia is well documented, appears feasible even in older patients, taking advantage of the new tools that could guarantee higher safety and efficacy standards.

## Data Availability

All data and images are available in CdC “Villa Verde” EP lab in Taranto (Italy).
